# Effect of a Combination of Prednisone or Mycophenolate Mofetil and Mesenchymal Stem Cells on Lupus Symptoms in MRL.*Fas*^lpr^ Mice

**DOI:** 10.1155/2018/4273107

**Published:** 2018-07-03

**Authors:** Hong Kyung Lee, Ki Hun Kim, Hyung Sook Kim, Ji Sung Kim, Jae Hee Lee, Ayoung Ji, Kyung Suk Kim, Tae Yong Lee, In Young Chang, Sang-Cheol Bae, Jin Tae Hong, Youngsoo Kim, Sang-Bae Han

**Affiliations:** ^1^College of Pharmacy, Chungbuk National University, Cheongju, 28160 Chungcheongbuk-do, Republic of Korea; ^2^Corestem Inc., Seongnam, 13486 Gyeonggi-do, Republic of Korea; ^3^Hanyang University Hospital for Rheumatic Diseases, Seoul 04763, Republic of Korea

## Abstract

The combination of immunosuppressants and mesenchymal stem cells (MSCs) is a promising therapeutic strategy for systemic lupus erythematosus, since this approach reduces doses of immunosuppressants while maintaining the same therapeutic outcome. However, it is unavoidable for MSCs to be exposed to immunosuppressants. Here, we examined the combination effect of prednisone (PD) or mycophenolate mofetil (MMF) and MSCs. We showed that PD or MMF in combination with MSCs showed better therapeutic effect than single therapy in lupus-prone MRL.*Fas*^lpr^ mice, as assessed by using the following readouts: prolongation of survival, decrease in anti-dsDNA and total IgG levels in serum, decrease in cytokine gene expression in spleen cells, and decrease in inflammatory cell infiltration into the kidney. *In vitro*, immunosuppressants and MSCs inhibited T cell proliferation in a synergistic manner. However, immunosuppressants did not affect MSC viability and functions such as TGF-*β*1 and PGE_2_ production, migration, and immunosuppressive capacity. In summary, our study demonstrates that a combination of immunosuppressants and MSCs is a good strategy to reduce the side effects of PD and MMF without the loss of therapeutic outcome.

## 1. Introduction

Systemic lupus erythematosus (SLE) is a multiorgan disease characterized by abnormalities of T and B cells and production of autoantibodies [1]. Nephritis occurs in 40–75% of patients and is associated with increased morbidity and mortality. SLE has been traditionally treated using immunosuppressants, such as cyclophosphamide, prednisone (PD), and mycophenolate mofetil (MMF) [1]. PD, which is metabolized to prednisolone *in vivo*, acts through a genomic and a nongenomic pathway. In the genomic pathway, PD binds cytosolic receptors to form a regulatory complex, which translocates to the nucleus, binds to DNA, and represses the transcription of many inflammatory mediators [2]. PD also activates the expression of genes associated with osteoporosis, cataracts, hyperglycemia, coronary heart disease, and cognitive impairment, which are thought to be responsible for the most serious adverse effects of PD [3]. In the nongenomic pathway, PD interacts with diverse proteins in cytosol and membranes [4]. PD is a strong anti-inflammatory agent that inhibits proliferation of and inflammatory mediator production by immune cells [2]. MMF is a prodrug of mycophenolic acid (MPA), an inhibitor of inosine monophosphate dehydrogenase (IMPDH), which is the rate-limiting enzyme in the de novo synthesis of guanosine nucleotides [5]. MMF is a potent cytostatic agent and strongly inhibits the proliferation of T and B cells [5].

Mesenchymal stem cells (MSCs) alone or in combination with the currently used immunosuppressive therapy have been investigated as a promising therapeutic strategy for SLE [6]. MSCs can be isolated from various tissues including the skeletal muscle, umbilical cord blood, adipose tissue, and bone marrow (BM); they are self-renewable and can differentiate into osteocytes, myocytes, adipocytes, and other cell types [7, 8]. They adhere under normal culture conditions and express CD73, CD90, and CD105, but not CD34 or CD45 [9]. Interestingly, MSCs can regulate immune responses through soluble mediators and cell-cell contacts: they inhibit T cell proliferation and cytokine production, decrease B cell proliferation and antibody secretion, inhibit the maturation and functions of dendritic cells, and reduce proliferation and functions of NK cells but enhance the activity of Treg cells [8].

A combination of MSCs and immunosuppressants significantly ameliorated symptoms in SLE patients [10]. Yet, an important question remains: do immunosuppressants interfere with MSC functions in combination therapy? MSC functions might be inhibited by immunosuppressants, since they express the molecular targets of immunosuppressants. To address this issue, we examined the effects of an immunosuppressant-MSC combination on lupus-prone MRL.*Fas*^lpr^ mice and investigated the effects of PD and MMF on survival, soluble mediator production, migration, and immunosuppressive capacity of human BM-derived MSCs.

## 2. Materials and Methods

### 2.1. Mesenchymal Stem Cells and Immunosuppressants

Human BM-derived MSCs were obtained from Corestem Inc. (Seoul, Korea). In brief, BM was aspirated from the posterior iliac crest of healthy donors and mononuclear cells were purified by density gradient centrifugation [11]. These cells were cultured in CSMB-A06 medium (Corestem Inc.) containing 10% fetal bovine serum (BD Biosciences, Franklin Lakes, NJ, USA), 2.5 mM l-glutamine, and penicillin/streptomycin (WelGene, Gyeonggi, Korea) in a 5% CO_2_ incubator at 37°C for 3–5 passages. After washing out nonadherent cells, the adherent cells retained the canonical phenotype of MSCs (CD29^+^CD44^+^CD73^+^CD105^+^CD90^+^CD34^−^CD45^−^HLA-DR^−^) and were used in the experiments. All human MSC experiments were approved by the Institutional Review Board of Hanyang University Hospital and were carried out in accordance with their approved guidelines; all participants provided written informed consent. PD, prednisolone (PDL), and MPA were purchased from Sigma-Aldrich (St. Louis, MO, USA). MMF was purchased from Kyongbo Pharmaceutical Co. Ltd. (Asan, Korea).

### 2.2. MRL.*Fas*^lpr^ Mouse Model

MRL.*Fas*^lpr^ mice were purchased from the Jackson Laboratory (Bar Harbor, ME, USA). Mice were housed in specific pathogen-free conditions at 21–24°C and 40%–60% relative humidity under a 12 h light/dark cycle. In each experiment, female mice were divided into four groups (6 mice per group): control (vehicle), PD (0.5 mg/kg) or MMF (50 mg/kg), MSC (4 × 10^4^ cells/injection), and a combination of PD or MMF and MSCs. MSCs were injected intravenously once at the age of 12 weeks. PD was injected intraperitoneally once a week from 10 to 16 weeks of age. MMF was administered orally twice a week from 10 to 19 weeks of age. Survival rate and body weight were measured weekly. The serum levels of anti-dsDNA IgG and total IgG were measured by using an anti-dsDNA IgG ELISA kit (Alpha Diagnostic International, San Antonio, TX, USA) and a total IgG ELISA kit (eBioscience, San Diego, CA, USA) [11]. All animal experiments were approved by the Chungbuk National University Animal Experimentation Ethics Committee and were carried out in accordance with their approved guidelines.

### 2.3. Flow Cytometry

Spleens were isolated from MRL.*Fas*^lpr^ mice at 25 weeks of age, and immune cell composition was analyzed by flow cytometry. Spleen cells were stained with the following monoclonal antibodies in 0.5% BSA/PBS at 4°C for 15 min: FITC-labeled anti-B220, APC-labeled anti-CD3, PE-labeled anti-CD4, FITC-labeled anti-CD8, FITC-labeled anti-CD11c, PE-labeled anti-CD11b, APC-labeled anti-CD138, FITC-labeled anti-IgG1 (BD Biosciences, San Jose, CA, USA), FITC-labeled anti-CD4, and PE-labeled anti-Foxp3 (eBioscience). Data were collected using FACSCalibur and analyzed with CellQuest Pro software (BD Biosciences).

### 2.4. RT-PCR

Total RNA was isolated from spleen cells of MRL.*Fas*^lpr^ mice using TRIzol reagent (Thermo Fisher Scientific, Waltham, MA, USA). RNA was quantified using a spectrophotometer and stored at −80°C at a concentration of 1 mg/ml [12]. Reverse transcription polymerase chain reaction (RT-PCR) was performed to determine the relative quantities of mRNAs for IL-1*β*, TNF-*α*, IFN-*γ*, and IL-12 and housekeeping gene *β*-actin. cDNA was synthesized from 3 *μ*g total RNA using an RT kit (Bioneer, Daejeon, Korea). All of the PCRs were performed with a GenePro thermal cycler (Bioer, Hangzhou, China) by using a final volume of 50 *μ*l containing reaction buffer (1 mM Tris-HCl, 5 mM KCl, and 0.1% Triton X-100), 2 mM MgCl_2_, 200 *μ*M deoxynucleoside triphosphates, 0.2 *μ*M each primer, 1.25 U of *Taq* thermostable polymerase (Promega), and template cDNA. After an initial incubation at 95°C for 2 min, temperature cycling was begun. The cycles consisted of 20 sec of denaturation at 94°C, 30 sec of primer annealing at 56°C, and 30 sec of primer extension at 72°C for 30 cycles. All PCR products were analyzed by electrophoresis on 1% agarose gels and visualized by staining with ethidium bromide staining (0.5 *μ*g/ml). The primer sequences were as follows: IL-1*β*, sense, 5′-ATG GCA ATG TTC CTG AAC TCA ACT-3′, antisense, 5′-CAG GAC AGG TAT AGA TTC TTT CCT TT-3′; IFN-*γ*, sense, 5′-AGC GGC TGA CTG AAC TCA GAT TGT AG-3′, antisense, 5′-GTC ACA GTT TTC AGC TGT ATA GGG-3′; TNF-*α*, sense, 5′-AGG TTC TGT CCC TTT CAC TCA CTG-3′, antisense, 5′-AGA GAA CCT GGG AGT CAA GGT A-3′; IL-12, sense, 5′-AGA GGT GGA CTG GAC TCC CGA-3′, antisense, 5′-TTT GGT GCT TCA CAC TTC AG-3′; and *β*-actin, sense, 5′-TGG AAT CCT GTG GCA TCC ATG AAA C-3′, antisense 5′-TAA AAC GCA GCT CAG TAACAG TCC G-3′.

### 2.5. Immunohistochemistry

Kidneys were also isolated from MRL.*Fas*^lpr^ mice at 25 weeks of age, fixed with 4% formalin, and immersed in PBS. After dehydration with ethanol and xylene, the tissues were embedded in paraffin and cut into 4 *μ*m sections. After deparaffinization and dehydration, sections were heated in a microwave oven (650 W, 20 min) for antigen retrieval, after which endogenous peroxidase activity was blocked by 3% hydrogen peroxide [13]. Thereafter, sections were incubated with the primary antibodies: goat antibody against mouse CD19 (diluted 1 : 100; Biolegend, San Diego, CA, USA), CD3 (diluted 1 : 100, Santa Cruz Biotechnology, Dallas, TX, USA), F4/80 (diluted 1 : 100, Santa Cruz Biotechnology), CD209b (diluted 1 : 100, Santa Cruz Biotechnology), and Foxp3 (diluted 1 : 50, Abcam, Cambridge, England) at 4°C overnight. Sections were then incubated with secondary antibody, anti-goat IgG conjugated with horseradish peroxidase (Vector Laboratories, Burlingame, CA, USA), for 1 h at room temperature. Signals were developed with a two-component high-sensitivity diaminobenzidine chromogenic substrate (Vector Laboratories) for 10 min and counter stained with hematoxylin.

### 2.6. Viability Test

Effect of immunosuppressants on MSC viability was determined with XTT assay. MSCs were seeded in a 96-well plate at 1 × 10^4^ cells/well and incubated with immunosuppressants for 24 h. XTT reagent (50 *μ*l; Roche, Mannheim, Germany) was then added, cells were incubated for an additional 6 h, and absorbance was read at 450 nm using a SpectraMax 190 Microplate Reader (Molecular Devices, Sunnyvale, CA, USA). The levels of TGF-*β* and PGE_2_ in culture medium were determined by ELISA kits (R&D Systems, Minneapolis, MN, USA).

### 2.7. Migration Assay

Effect of immunosuppressants on MSC migration was examined in transwell plates with 8 *μ*m pore filters (Corning, Cambridge, MA, USA) [14]. Upper wells were precoated with 0.1% gelatin (Sigma-Aldrich) for 2 h at 37°C. Upper wells were loaded with 2 × 10^4^ MSCs in 200 *μ*l of medium and the lower wells with 500 *μ*l of medium containing 10% fetal bovine serum and 100 ng/ml CXCL10 (R&D Systems). After 24 h, nonmigrated cells on the upper side of the filters were removed and the membranes were fixed in 10% formalin. MSCs that migrated to the lower side of the filter were stained with 0.5% crystal violet for 10 min and were counted under an inverted light microscope.

### 2.8. T Cell Function Assay

MSCs were pretreated with immunosuppressants for 24 h, harvested, washed three times with medium, and seeded at 1 × 10^4^ cells/well in a 96-well plate. T cells were purified from spleen cells of MRL*.Fas*^lpr^ mice by a negative depletion method using biotinylated antibodies specific for B220, GR-1, and CD11c (BD Biosciences) and Dynabeads M-280 Streptavidin (Thermo Fisher Scientific) [11]. Purity was typically >90%. Splenic T cells were added at 1 × 10^5^ cells/well, and concanavalin A (ConA; 1 *μ*g/ml) was added to activate the cells. After 52 h incubation, T cells were pulsed with 1 *μ*Ci ^3^H-thymidine/well (113 Ci/nmol; NEN, Boston, MA, USA) and harvested 18 h later. Incorporated radioactivity was measured using a Microbeta scintillation counter (Wallac, Turku, Finland) [15]. In some experiments, culture medium was collected after 72 h incubation and the level of IFN-*γ* was quantified by an ELISA kits (R&D Systems). We also examined the effect of a combination of MSCs and immunosuppressants on T cell proliferation. MSCs and/or immunosuppressants were added to splenic cells and ConA (1 *μ*g/ml) was added to induce T cell proliferation. After 52 h incubation, cells were pulsed with 1 *μ*Ci ^3^H-thymidine/well and harvested 18 h later. Incorporated radioactivity was measured using a Microbeta scintillation counter.

### 2.9. Statistical Analysis

Data are the mean ± SEM of at least three independent experiments each performed in triplicate (*in vitro*) or six mice (*in vivo*); *p* values were calculated using one-way ANOVA in GraphPad Prism software (GraphPad, San Diego, CA, USA).

## 3. Results

### 3.1. Effect of a Combination of PD and MSCs in Lupus-Prone MRL.*Fas*^lpr^ Mice

**Figure 1:**
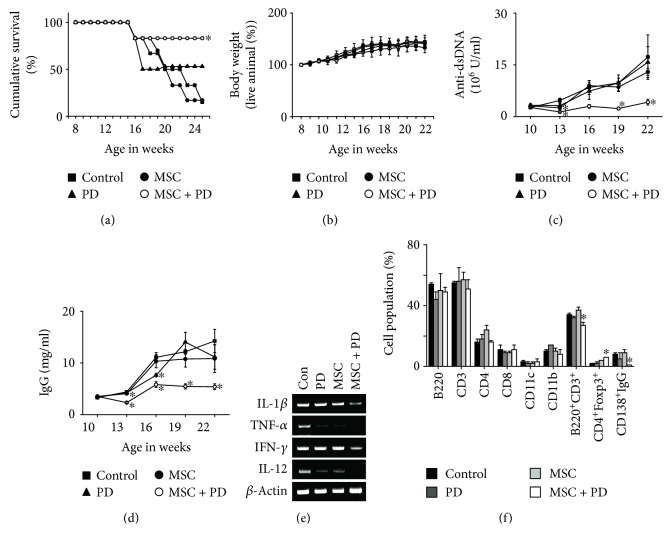
Effect of a combination of PD and MSCs in lupus-prone MRL.*Fas*^lpr^ mice. Mice were intravenously injected with MSCs once at 12 weeks of age and with PD intraperitoneally once a week from 10 to 16 weeks of age. Survival (a) and body weight (b) were measured weekly. The levels of anti-dsDNA IgG (c) and total IgG (d) in serum were measured every 3 weeks. (e) Total RNA was purified from spleen cells, which were isolated from surviving mice at 20 weeks of age, and the expression of IL-1*β*, TNF-*α*, IFN-*γ*, and IL-12 was examined by RT-PCR. (f) Subset ratios were analyzed by flow cytometry. ^∗^*p* < 0.01 versus control.

In our preliminary study, we observed that only 10% of untreated mice survived until 24 weeks of age, but >80% of mice treated with PD (1 mg/kg) and >70% of mice treated with MSCs (4 × 10^5^ cells/injection) lived until this age. Thus, we injected lower doses of PD (0.5 mg/kg) and MSCs (4 × 10^4^ cells/injection) to determine the effect of their combination. The combination of PD and MSCs significantly prolonged survival compared to each therapy alone: 90% of the mice receiving PD and MSCs survived up to 25 weeks of age, whereas only 10% of control and MSC-treated mice and 50% of PD-treated mice survived (Figure 1(a)). None of the therapies affected body weight (Figure 1(b)), and no untoward effects were noted. The serum level of anti-dsDNA IgG (Figure 1(c)) and total IgG antibodies (Figure 1(d)) decreased in mice treated with the PD-MSC combination compared to each therapy alone.

**Figure 2:**
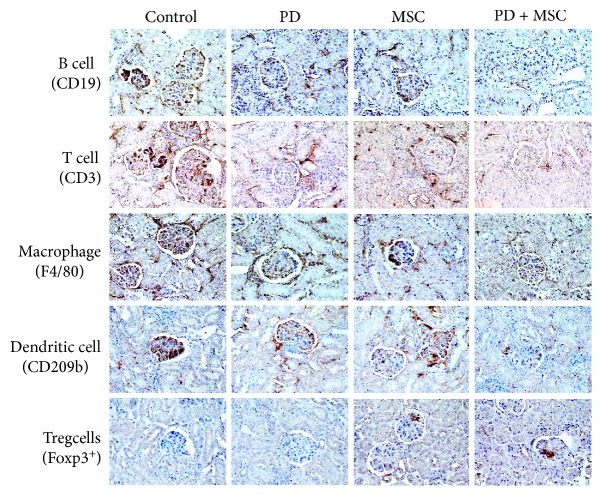
Representative images of immunohistochemical staining of kidneys after treatment with a PD-MSC combination. MRL*.Fas*^lpr^ mice were intravenously injected with MSCs once at 12 weeks of age and with PD intraperitoneally once in a week from 10 to 16 weeks of age. Kidneys were isolated from surviving mice at 20 weeks of age. Kidney sections were stained with antibodies against CD19 (B cells), CD3 (T cells), F4/40 (macrophages), CD209b (dendritic cells), and Foxp3^+^ (Treg cells).

We isolated spleen cells from surviving 25-week-old mice. The expression of all inflammatory cytokines tested (IL-1*β*, TNF-*α*, IFN-*γ*, and IL-12) decreased in the spleens of mice treated with the PD-MSC combination compared to each therapy alone (Figure 1(e)), whereas the frequency of CD4^+^Foxp3^+^ Treg cells increased and that of CD138^+^IgG^+^ plasma cells decreased (Figure 1(f)). No infiltration of B cells, T cells, macrophages, dendritic cells, or Treg cells into the kidney was detected before disease onset (5 weeks of age; data not shown); their infiltration strongly increased with disease progression (25 weeks of age) and decreased in mice treated with a combination of PD and MSCs (Figure 2). The infiltration of Treg cells decreased with the progression of disease in control mice, and this trend was reversed by the combination of PD and MSCs (Figure 2). Single therapies did not affect immune cell infiltration into the kidney due to suboptimal dosage of PD or MSCs. These data show that treatment with a combination of PD and MSCs strongly ameliorates the development of lupus symptoms in MRL*.Fas*^lpr^ mice compared to single therapies.

### 3.2. Combination Effect of MMF and MSCs in MRL.*Fas*^lpr^ Mice

**Figure 3:**
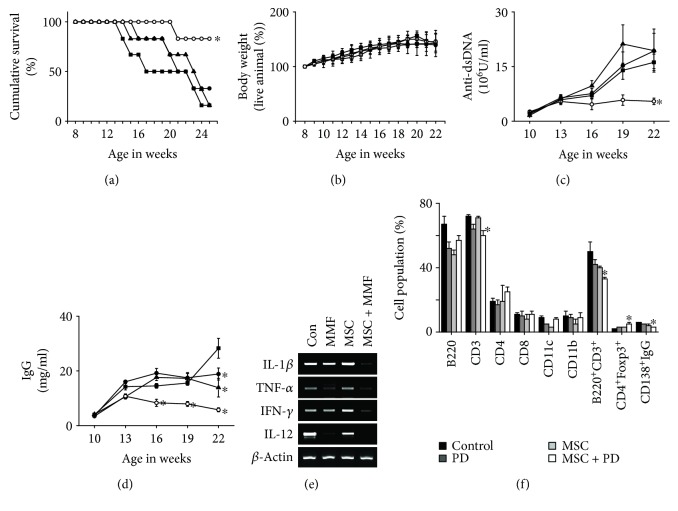
Effect of a combination effect of MMF and MSCs in lupus-prone MRL.*Fas*^lpr^ mice. Mice were intravenously injected with MSCs once at 12 weeks of age and with MMF orally twice a week from 10 to 19 weeks of age. Survival (a) and body weight (b) were measured weekly. The levels of anti-dsDNA IgG (c) and total IgG (d) in serum were measured every 3 weeks. (e) Total RNA was purified from spleen cells, which were isolated from surviving mice at 20 weeks of age, and the expression of IL-1*β*, TNF-*α*, IFN-*γ*, and IL-12 was examined by RT-PCR. (f) Subset ratios were analyzed by flow cytometry. ^∗^*p* < 0.01 versus control.

**Figure 4:**
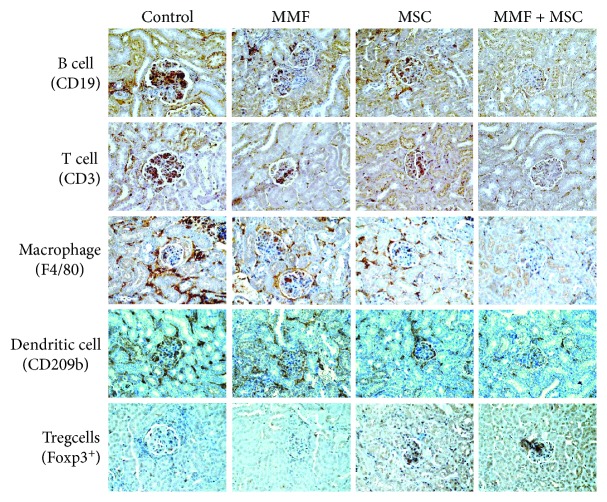
Representative images of immunohistochemical staining of kidneys after treatment with a MMF-MSC combination. MRL*.Fas*^lpr^ mice were intravenously injected with MSCs once at 12 weeks of age and with MMF orally twice a week from 10 to 19 weeks of age. Kidneys were isolated from surviving mice at 20 weeks of age. Kidney sections were stained with antibodies against CD19 (B cells), CD3 (T cells), F4/40 (macrophages), CD209b (dendritic cells), and Foxp3^+^ (Treg cells).

Next, we examined the therapeutic activity of a combination of MMF and MSCs. Since 90% of the mice receiving MMF at 100 mg/kg survived up to 24 weeks of age, we injected lower doses of MMF (50 mg/kg). A combination of MMF and MSCs significantly prolonged survival (by 90%) at 25 weeks of age, which was higher than single therapy of MMF or MSCs (Figure 3(a)). None of the therapies affected body weight (Figure 3(b)). The serum level of anti-dsDNA (Figure 3(c)) and total IgG antibodies (Figure 3(d)) decreased by the combination therapy compared to each therapy alone. At 25 weeks of age, we isolated spleen cells from several surviving mice. Combination therapy decreased the expression of all inflammatory cytokines tested (IL-1*β*, TNF-*α*, IFN-*γ*, and IL-12) in comparison with each therapy alone (Figure 3(e)). The frequency of CD4^+^Foxp3^+^ Treg cells increased and that of CD138^+^IgG^+^ plasma cells in the spleen was lower after combination therapy than after each therapy alone (Figure 3(f)). Immunohistological data showed that the infiltration of B cells, T cells, macrophages, dendritic cells, and Treg cells into the kidney was decreased by combination therapy in comparison with each therapy alone (Figure 4). The infiltration of Treg cells decreased with the progression of disease in control mice, and this trend was reversed by the combination therapy (Figure 4). These data show that treatment with a combination of MMF and MSCs strongly ameliorates the development of lupus symptoms in MRL*.Fas*^lpr^ mice compared to single therapies.

### 3.3. Effect of Combinations of Immunosuppressants and MSCs In Vitro

**Figure 5:**
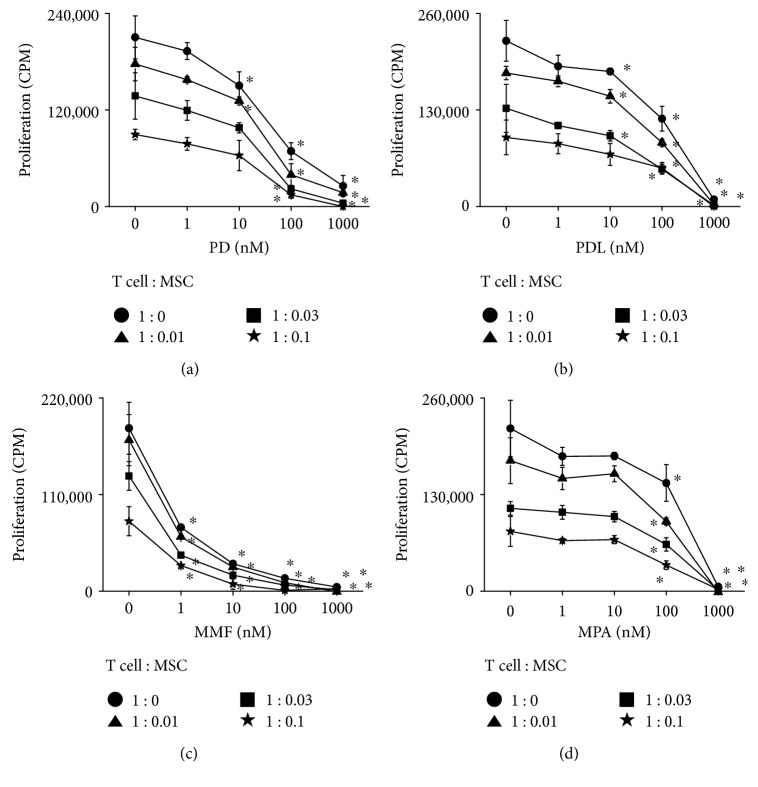
Combination effect of immunosuppressants and MSCs *in vitro.* T cells were loaded into a 96-well plate at 1 × 10^5^ cells/well. MSCs were loaded at 0.01, 0.03, or 0.1 × 10^4^ cells/well, or were omitted. Cells were treated with PD (a), PDL (b), MMF (c), or MPA (d). Concanavalin A was added at 1 *μ*g/ml and T cell proliferation was measured 72 h later. ^∗^*p* < 0.01 versus control.

Next, we examined the effects of combinations of immunosuppressants and MSCs on T cell proliferation *in vitro*. PD alone inhibited ConA-induced T cell proliferation with an IC_50_ of 37.5 nM and MSCs with 6819 cells/injection; their combination synergistically inhibited T cell proliferation (Figure 5(a)). PDL, a metabolite of PD, inhibited T cell proliferation with an IC_50_ of 8.2 nM, and its combination with MSCs synergistically inhibited T cell proliferation (Figure 5(b)). MMF alone inhibited T cell proliferation with an IC_50_ of 0.4 nM (Figure 5(c)); MPA, a metabolite of MMF, had an IC_50_ of 149.6 nM (Figure 5(d)). MMF or MPA in combination with MSCs synergistically inhibited T cell proliferation (Figures 5(c) and 5(d)). These data confirm that immunosuppressants in combination with MSCs synergistically inhibit T cell proliferation [16].

### 3.4. Direct Effect of Immunosuppressants on MSC Functions

**Figure 6:**
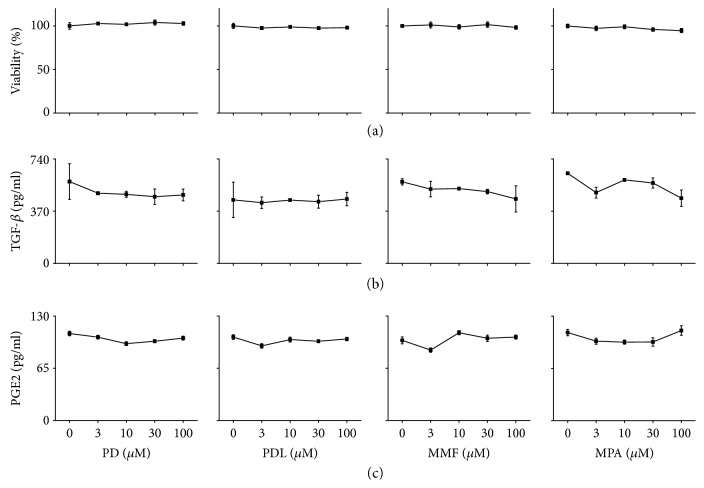
Direct effect of immunosuppressants on MSC functions *in vitro.* MSCs were loaded into a 96-well plate at 1 × 10^4^ cells/well and treated with immunosuppressants for 24 h. Viability was measured by XTT assay (a) and the levels of TGF-*β*1 (b) and PGE_2_ (c) in culture medium were measured by ELISA.

**Figure 7:**
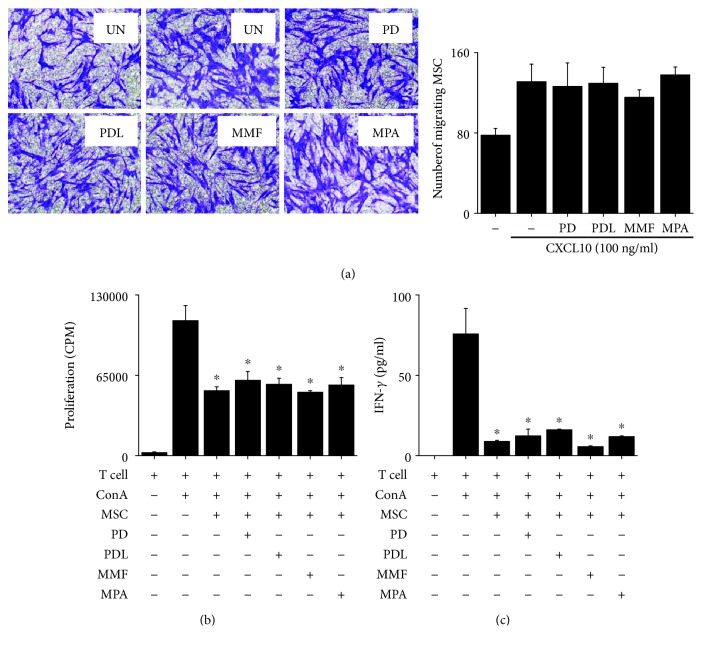
Effect of immunosuppressants on MSC functions *in vitro*. MSCs were pretreated with 100 *μ*M of PD, PDL, MMF, or MPA for 24 h. MSCs (2 × 10^4^ cells/well) were added to the upper chamber and allowed to migrate to the lower chamber containing CXCL-10 (100 ng/ml). MSCs that migrated to the lower side of inserts were stained with crystal violet (a). Immunosuppressant-pretreated MSCs (0.1 × 10^4^ cells/well) were mixed with T cells (1 × 10^5^ cells/well) in a 96-well plate. Concanavalin A (ConA) was added at 1 *μ*g/ml, and the proliferation of (b) and IFN-*γ* production (c) by T cells were measured 72 h later. ^∗^*p* < 0.01 versus control.

We reasoned that immunosuppressants might affect MSC functions and examined this possibility. In our preliminary experiments, none of the immunosuppressants (PD, PDL, MMF, and MPA) affected MSC functions at concentration below 1 *μ*M. At higher concentrations (3–100 *μ*M), all immunosuppressants inhibited the proliferation of and IFN-*γ* production by ConA-activated T cells (data not shown), but did not affect MSC viability (Figure 6(a)), TGF-*β*1 production (Figure 6(b)), or PGE_2_ production (Figure 6(c)). These data suggest that immunosuppressants up to 100 *μ*M do not affect the soluble factor production by MSCs. Next, we examined whether immunosuppressant-treated MSCs had normal functions. MSCs treated with 100 *μ*M immunosuppressants for 24 h showed the same potency of migration toward CXCL10 as control MSCs (Figure 7(a)). Immunosuppressant-treated MSCs similarly inhibited the proliferation of (Figure 7(b)) and IFN-*γ* production (Figure 7(c)) by ConA-activated T cells. Overall, these data suggest that none of the immunosuppressants interfered with MSC functions.

## 4. Discussion

Immunosuppressive drugs are widely used to treat SLE, but their clinical use is often limited by harmful side effects. The combined application of immunosuppressants and MSCs offers a promising alternative approach, which will decrease the doses of immunosuppressants with maintaining the outcome of therapy. Here, we show that a combination of a low dose of PD or MMF and MSCs ameliorates lupus symptoms in MRL.*Fas*^lpr^ mice to the same extent as a high dose of PD or MMF and that this combination also inhibits T cell proliferation in a synergistic manner. Potentially, a critical drawback of this combination therapy might be unwanted effects of PD or MMF on MSCs. In the combination approach, MSCs are exposed to diverse immunosuppressants. Although the molecular targets of these drugs are expressed in both lymphocytes and MSCs, they responded differently: PD or MMF strongly inhibited lymphocyte functions, but not MSC viability and functions, such as TGF-*β*1 and PGE_2_ production, migration, and immunosuppressive capacity. These data suggest that combination therapy of MSCs and a low dose of PD or MMF is a good strategy to attenuate SLE progression.

Our data extend previous studies that have examined the effects of combinations of MSCs and the calcineurin inhibitor cyclosporine A, FK506-binding protein inhibitor tacrolimus, mTOR inhibitor rapamycin, and IMPDH inhibitor MMF [17–19]. Most studies showed that these compounds did not affect the viability and immunosuppressive capacity of MSCs [17, 18]. However, several studies showed inconsistent results: tacrolimus increased MSC inhibitory capacity [17], cyclosporine A increased the inhibition of lymphocyte proliferation by MSCs [17] and enhanced MSC viability [20], and MMF promoted the inhibitory activity of MSCs, whereas cyclosporine A, tacrolimus, and rapamycin antagonized it [21]. Glucocorticoids, such as dexamethasone and PD, are widely used in SLE patients because of their potent anti-inflammatory properties, although they have severe side effects [18]. Dexamethasone enhances human growth factor production by MSCs [18]. PD increases indoleamine 2,3-dioxygenase production by MSCs without affecting other immunosuppressive capacities [18]. Several studies also showed the efficacy of combinations of immunosuppressants and MSCs [19, 22, 23]. Cotreatment with one of the immunosuppressants and MSCs strongly inhibited Th1 and Th17 cell functions (proliferation, inflammatory cytokine production, and differentiation) but promoted Th2 and Treg cell functions [19]. A combination of MMF and MSCs prolonged allograft survival [22, 23]. In the present study, we observed that PD or MMF in combination with MSCs synergistically inhibited T cell functions without affecting MSC functions. Some discrepancies between our data and those from several previous studies might be due to the diversity of MSC sources, human donor conditions, culture conditions, or experimental conditions, and further studies are required.

A critical question is why PD or MMF does not affect MSC functions. Because MMF inhibits lymphocyte proliferation by inhibiting IMPDH, which is also expressed in MSCs; MMF could be expected to affect MSC functions [2, 5]. The absence of the effect of MMF on MSC viability or functions might be due to the slow proliferation of MSCs: the reported mean doubling time of bone marrow-derived MSCs is 40–60 h and that of adipose tissue-derived MSCs is 24–45 h [24]. The mean doubling time of our BM-derived MSCs was 50 h (data not shown), suggesting that treatment with MMF for 24–48 h was not sufficient to affect MSC proliferation. Glucocorticoids including PD play an essential role in inducing MSC differentiation to adipocytes [25]. PD binds to the glucocorticoid receptor in the cytosol and forms a regulatory complex on response elements in the target gene encoding myostatin [26]. Myostatin is sufficient to elicit the adipocyte fate decision [27]. However, nothing is known about the effect of glucocorticoids including PD on MSC functions. Our data show that PD does not affect MSC viability or functions, when the cells are treated for 24–48 h. In the future, we will intensively study whether long-term and repeated exposure to immunosuppressants will affect MSC functions, since SLE patients need long-term and repeated treatment with immunosuppressants [19].

Further study will be required to examine the expression levels of inflammatory cytokines in the kidney of mice treated with a combination of MSCs and immunosuppressants. The deposit of immune complex in the kidney activates mesangial and endothelial cells, which produce various types of cytokines, such as IL-1, IL-6, IL-12, IFN-*γ*, and TNF-*α* [28]. Single-cell analysis on mesangial cells, endothelial cells, and podocytes will give valuable information to understand the therapeutic mechanism of MSCs for lupus nephritis. It will be also interesting to examine the chemokine expression levels in nephritic kidneys. The chemokines CCL2, CCL3, CCL5, CXCL10, CXCL12, CXCL13, and CX3CL1 are expressed in the nephritic kidney of lupus-prone mice and SLE patients and increase inflammatory cell infiltration into the kidney [29]. Further clinical studies will be also required to address whether PD or MMF affects MSC functions in patients. In the present study, we transplant human MSCs and inject immunosuppressants to MRL.*Fas*^lpr^ mice, which lack *Fas* and spontaneously develop a lupus-like disease [11]. Although these mice have been widely used in efficacy evaluation of human MSCs in the preclinical studies, xenogeneic human MSCs may induce rejection and adverse inflammation, which will affect lupus progression and therapeutic activity of MSCs. Although xenogeneic human MSCs may escape immune recognition and clearance in mice due to the low expression of MHC-I and no expression of MHC-II, a mouse model does not accurately reflect human condition [11].

The current immunosuppressive protocols for SLE are based on the administration of several immunosuppressants, each with a different mechanism. When combining immunosuppressants and MSCs, two points are needed to be considered. First, it is desirable to decrease the dose of steroids and immunosuppressants, since they have severe side effects. Second, maintaining the cell number and immunosuppressive capacity of MSCs should be taken into account, since enough MSCs should be used to achieve high therapeutic efficacy. Our data demonstrate that the dose of PD or MMF can be lowered by combining it with MSCs for the treatment of SLE and PD or MMF does not affect MSC functions, providing an important insight on the application of this combination therapy for SLE patients.

## Data Availability

The data used to support the findings of this study are available from the corresponding author upon request.
